# Flow-Seq Evaluation of Translation Driven by a Set of Natural *Escherichia coli* 5′-UTR of Variable Length

**DOI:** 10.3390/ijms232012293

**Published:** 2022-10-14

**Authors:** Ekaterina S. Komarova, Anna N. Slesarchuk, Maria P. Rubtsova, Ilya A. Osterman, Alexey E. Tupikin, Dmitry V. Pyshnyi, Olga A. Dontsova, Marsel R. Kabilov, Petr V. Sergiev

**Affiliations:** 1 Institute of Functional Genomics, Lomonosov Moscow State University, 119992 Moscow, Russia; 2 Department of Chemistry, Lomonosov Moscow State University, 119991 Moscow, Russia; 3 Center for Life Sciences, Skolkovo Institute of Science and Technology, Skolkovo, 143025 Moscow, Russia; 4 Institute of Chemical Biology and Fundamental Medicine, Siberian Branch of the Russian Academy of Sciences, 630090 Novosibirsk, Russia; 5 Shemyakin-Ovchinnikov Institute of Bioorganic Chemistry, Russian Academy of Sciences, 119992 Moscow, Russia; 6 Belozersky Institute of Physico-Chemical Biology, Lomonosov Moscow State University, 119992 Moscow, Russia

**Keywords:** ribosome, translation, Flow-seq, untranslated region, translation efficiency

## Abstract

Flow-seq is a method that combines fluorescently activated cell sorting and next-generation sequencing to deduce a large amount of data about translation efficiency from a single experiment. Here, we constructed a library of fluorescent protein-based reporters preceded by a set of 648 natural 5′-untranslated regions (5′-UTRs) of *Escherichia coli* genes. Usually, Flow-seq libraries are constructed using uniform-length sequence elements, in contrast to natural situations, where functional elements are of heterogenous lengths. Here, we demonstrated that a 5′-UTR library of variable length could be created and analyzed with Flow-seq. In line with previous Flow-seq experiments with randomized 5′-UTRs, we observed the influence of an RNA secondary structure and Shine–Dalgarno sequences on translation efficiency; however, the variability of these parameters for natural 5′-UTRs in our library was smaller in comparison with randomized libraries. In line with this, we only observed a 30-fold difference in translation efficiency between the best and worst bins sorted with this factor. The results correlated with those obtained with ribosome profiling.

## 1. Introduction

Translation contributes a several orders of magnitude difference to gene expression efficiency [1]. The elucidation of the principles behind translation efficiency is paramount for biotechnology, as well as for our understanding of cell functioning. A number of methods have been developed to decipher translation efficiency for both naturally created [2,3,4] and reporter [1,5,6,7,8] mRNAs, such as ribosome profiling and Flow-seq. The latter method relies on the creation of a large library of reporter constructs, where the fluorescent protein gene is placed under the control of specific regulatory sequences, such as promoters, ribosome binding sites, codon-biased areas, etc. Usually, artificial libraries of control elements are designed to be of the same length. Even if natural control elements are heterogenous in their size, for the sake of synthesis simplification, same-size areas of interest are generally selected for library construction [6].

Here, we challenged this pipeline and created a set of reporters based on 648 natural 5′-UTR sequences of *E. coli* genes, whose lengths varied from 2 to 60 nucleotides. This library was placed in front of the CER gene in a reporter construct, where a RFP gene, whose 5′-UTR was invariable, served as an internal control [9]. Using the Flow-seq method, we investigated the influence of the 5′-UTR regions of natural *E. coli* genes on translation efficiency.

## 2. Results

### 2.1. Selection of 5′-UTR Sequences and Reporter Library Creation

The selection of natural 5′-UTR sequences was conducted via two steps. First, we decided to restrict the 5′-UTR library to only the first genes of operons belonging to the *E. coli* BW25113 strain. The initial set of 1451 5′-UTR variants was restricted using the 60 nt length cut-off due to the oligonucleotide synthesis restrictions. Thus, the library including 713 natural 5′-UTRs with an inclusive length range of 2–60 nt was designed and represented to further PCR amplification with adaptor oligonucleotides containing endonuclease restriction sites necessary for the subsequent cloning into the vector. Of note, 5′-UTRs with a length of approximately 40 nt are the most frequently encountered among *E. coli* mRNAs [10,11].

**Figure 1:**
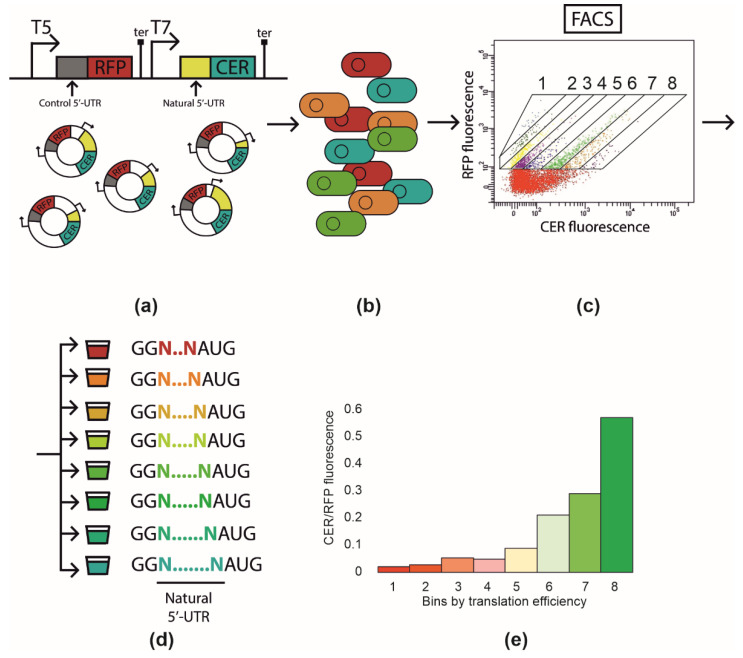
The principal scheme of the Flow-seq experiment. (**a**) Cloning of the DNA fragment containing natural 5′-UTR into pRFPCER reporter vector in front of *cer* gene while *rfp* gene retains its constant 5-UTR. (**b**) Transformation of the entire plasmid library into *E. coli* BL21 DE3 cells. (**c**) Fluorescence-activated cell sorting with CER/RFP fluorescence intensity ratio. Areas corresponding to fractions 1–8 are indicated. (**d**) Collection of cell fractions (1–8) according to CER/RFP fluorescence ratio. DNA extraction and amplification of 5′-UTR containing region followed by next-generation sequencing. (**e**) CER/RFP fluorescence ratio in the cells of fractions from 1 to 8 measured *en masse* with fluorimeter.

In order to analyze the influence of the natural 5′-UTRs sequence on translation efficiency, we used the plasmid encoding the red (RFP) and cerulean (CER) fluorescent proteins [6,9,12]. The reporter construction was based on two fluorescent protein genes, where the first one (*rfp*) was controlled by the constitutive T5 promoter and the second (*cer*) was under the control of the T7 promoter, both genes having their own terminators (Figure 1a). The 5′-UTRs of the *rfp* gene were identical in all plasmids in the library, allowing us to use the RFP as an internal control, whereas the 5′-UTRs of the *cer* gene, acting as a sensor, were presented with a set of 713 natural 5′-UTRs described earlier, appended only by two guanosine residues at the 5′ end for the sake of an efficient and uniform transcription. Natural 5′-UTR inserts with lengths of no longer than 60 nucleotides were cloned upstream the start codon of the *cer* gene at the HindIII and BamHI restriction sites, present also in the vector along with the gene providing resistance to ampicillin, for which further selection was carried out (Figure 1a).

### 2.2. Library Sorting and Sequencing

The created library was used to transform *E. coli* BL21 DE3 cells, which were plated on top of LB agar. Colonies of transformants were washed from the plate and pooled together. Cells were resuspended in saline and subjected to cell sorting. Eight fractions were selected using the ratio of the CER to RFP fluorescence intensity (Figure 1a–d), so that fraction eight contained the clones with the highest CER fluorescence. Cells of each fraction were used to measure the CER/RFP fluorescence ratio *en masse* to evaluate the efficiency of separation (Figure 1e). As expected, the CER fluorescence related to that of the RFP increased from fraction 1 to 8; the total difference between the ultimate fractions was ~30-fold. Sorted cells were used for the PCR amplification of the 5′-UTR region, which was subjected to further next-generation sequencing. Each 5′-UTR variant was attributed to a fraction, where the maximum number of reads corresponding to that variant was found (Supplementary Table S1). The resulting number of natural 5′-UTR sequences identified in all eight fractions and analyzed further was 648 out of the 713 subjected to cloning. We took the fractions as a proxy of translation efficiency, assuming that 5′-UTR sequence identity influenced mRNA stability to a lesser extent [6].

### 2.3. Properties of 5′-UTR Regions That Contribute to Translation Efficiency

**Figure 2:**
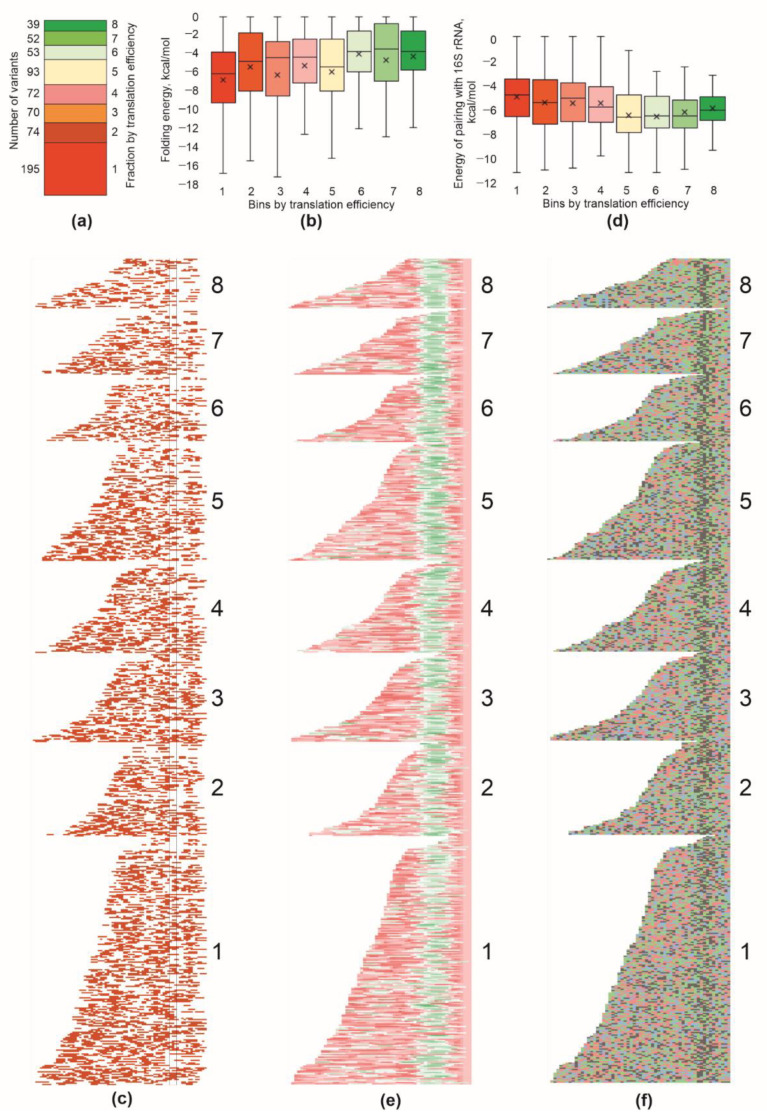
Properties of 5′-UTR sequences detrimental to translation efficiency. (**a**) Number of 5′-UTR variants that fell into each bin with translation efficiency (shown on the right side). Exact numbers of variants are indicated left to the bars. (**b**) Distribution of the folding energy of the mRNA region encompassing 5′-UTR and 16 nucleotides of the coding area in the bins sorted with translation efficiency. (**c**) Distribution of nucleotides involved in secondary structures. Each line corresponds to a particular mRNA part from the 5′-end to the position +16 to the beginning of the open reading frame. Location of the ATG start codon is shown with black lines. Red squares correspond to the nucleotides which were paired, white to the single-stranded nucleotides. All mRNAs were aligned with their 3′ edge. Sequences were sorted with translation efficiency (bins are indicated on the right side) and length (within each bin). (**d**) Distribution of the maximal interaction energy of the 5′-UTR with the anti-Shine–Dalgarno region of the 16S rRNA in the bins sorted with translation efficiency. (**e**) Distribution of SD sequences along 5′-UTRs tested. Each line corresponds to a particular mRNA 5′-UTR. Color indicates an energy of interaction within an 8 nt sliding window of mRNA with the 16S rRNA 3′ terminal region. Green corresponds to the efficient interaction, while red to no interaction. Principle of mRNA sorting was the same as in panel (**c**). (**f**) Nucleotide composition of the 5′-UTR sequences sorted with translation efficiency. Each line corresponds to a particular mRNA 5′-UTR. Blue squares correspond to C, red to U, green to A and grey to G. Principle of mRNA sorting was the same as in panels (**c**,**e**). See Supplementary Figure S1 for the presentation of the same data as sequence logo [13]. For panels (**b**–**d**), quartile ranges are shown as solid bars (25% to 50%) and thin lines (0% to 100%). Horizontal line corresponds to median, while cross to the average.

The translation efficiency of an individual mRNA is a subject of optimization rather than maximization due to the difference in the requirements for each protein in the cell. First of all, we assessed the number of 5′-UTR variants corresponding to each fraction (Figure 2a). Among our reporter library, the majority of 5′-UTR variants drove moderate expression, while the variants that boosted expression were found rarely, despite us having sampled fractions more densely for lower ratios of CER/RFP fluorescence.

The secondary structure of mRNAs can mask the ribosome binding site [6,9]. Thus, the folding energy of the mRNA region interacting with a ribosome during translation initiation is well-known to be an important factor governing translation efficiency. To this end, we analyzed the folding energy of the mRNA part encompassing 5′-UTR and the first 16 nucleotides of the coding region, which were likely to interact with a ribosome in the initiation process (Figure 2b,c). As expected, we observed, on average, weaker secondary structures in mRNAs translated more efficiently, especially in the region surrounding the start codon (Figure 2c). However, the range of secondary structure differences between the fractions was small. It goes in line with the idea that almost all natural 5′-UTRs have evolved to possess minimal secondary structures. We observed a minimal median energy of folding, ca. −6 kcal/mole, in the first fraction; however, this value was still incomparable with secondary structures that might drastically inhibit translation [8,9].

The best-known 5′-UTR element that determines translation efficiency is the Shine–Dalgarno sequence [14]. We analyzed a set of 5′-UTR regions composing our reporter set is search of the patches most likely to pair with the 3′-terminal part of 16S rRNA (Figure 2d,e). In agreement with the expectation, we found that 5′-UTRs possessing stronger SD pairings with the 16S rRNA (Figure 2d,e) drove, on average, a more efficient translation. No difference between the fractions in the location of the SD relative to the start codon was detected (Figure 2e).

We, additionally, checked for the presence of AU-rich translation enhancers [15] in the mRNA region upstream of the Shine–Dalgarno sequence (Figure 2f, Supplementary Figure S1). An increase in adenine and uridine nucleotide frequencies in a potential translation enhancer site was detected for all 5′-UTR bins grouped using translation efficiency, with a tendency for A-rich sequences to be prevalent for higher-efficiency 5′-UTRs, while U-rich ones for lower efficiency 5′-UTRs. This could be explained by the fact that we used natural 5′-UTRs that universally possess optimal necessary ribosome binding elements, while translation efficiency is adjusted by other means, such as, e.g., a codon frequency adjustment. Likewise, we could not see a clear difference in the AC nucleotide content between the fractions as was suggested earlier on the basis of a randomized 5′-UTR analysis [16].

**Figure 3:**
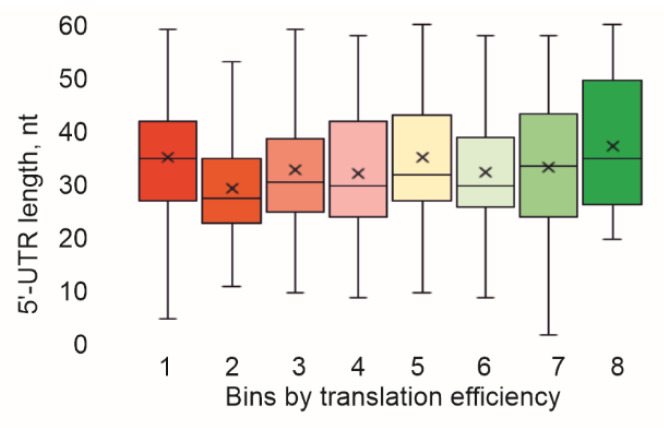
Influence of 5′-UTR length on translation efficiency. Shown is a distribution of the 5′-UTR length in the bins sorted using translation efficiency. Quartile ranges are shown as solid bars (25% to 50%) and thin lines (0% to 100%). Horizontal line corresponds to median, while cross to the average.

Additionally, we checked how the 5′-UTR lengths were distributed among the bins separated using translation efficiency (Figure 3). It appeared that the 5′-UTR length distribution was approximately the same for mRNAs with different translation efficiencies.

**Figure 4:**
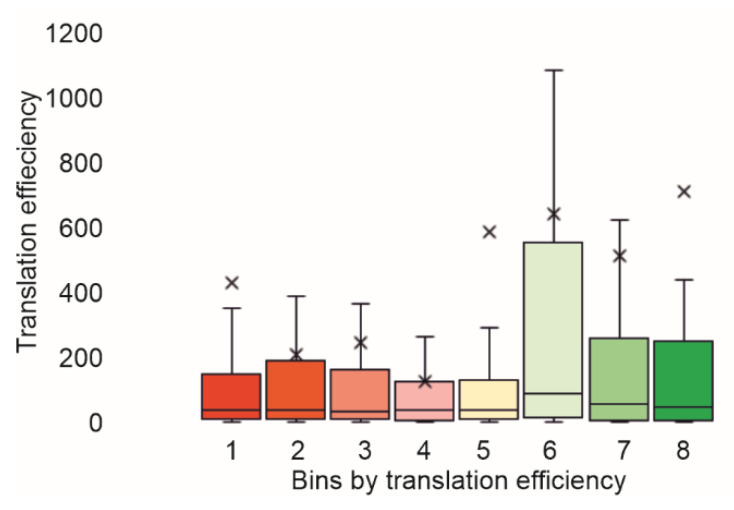
Comparison of translation efficiencies observed with Flow-seq and those obtained with ribosome profiling [19]. Bins sorted with translation efficiency were marked below the bars. *Y*-axis corresponds to the translation efficiency determined using ribosome profiling. Quartile ranges are shown as solid bars (25% to 50%). See Supplementary Figure S2 for comparison with other ribosome profiling experiments.

Finally, we compared the translation efficiencies observed by using Flow-seq, with those estimated using ribosome profiling [3,17,18,19]. As a result, we observed a decent correspondence between our data and those obtained in vivo (Figure 4, Supplementary Figure S2).

## 3. Discussion

Flow-seq is the method of choice for studying sequence elements that influence gene expression at all levels. This method allows one to test a wealth of natural [20] as well as artificial [6,16] regulatory elements in a standardized environment. In natural genes, all factors influence expression simultaneously, so it can be difficult to decipher the influence of any single factor. The majority of Flow-seq libraries has the drawback of using a standardized length of regulatory elements, which can alter natural secondary structures folded in, e.g., 5′-UTR, or remove potential distantly located control elements.

Here, we demonstrated the possibility to create a diverse library of reporter constructs possessing a range of lengths. In the current work, we assessed the influence of 5′-UTR on translation in *E. coli* while using a set of natural 5′-untranslated regions with lengths in the range of 2–60 nt. The obtained results fit the expectations and earlier experiment results with randomized 5′-UTR sequences well [6,7,16]. We observed an influence in secondary structures [21] and Shine–Dalgarno sequences on translation, although the variability of these parameters in the current dataset was much smaller in comparison with those in randomized 5′-UTR libraries. This is explained by the fact that we used a natural 5′-UTR set, which universally possesses elements that can positively influence protein synthesis and lacks, e.g., strong secondary structures that block translation. Accordingly, unlike the experiments with randomized [6] or artificially designed [9] 5′-UTRs, where the span of observed translation efficiencies reached four orders of magnitude, here, the difference in the efficiency between the marginal fractions was 30-fold at the maximum. In line with that, the complete randomization of 20-nucleotide-long 5′-UTRs that we used previously yielded only 0.03% of variants in the highest efficiency bin [6]. As we demonstrated here, natural variants of the 5′-UTR sequence demonstrated a much higher prevalence of efficient ones (6%). Logically, natural genes avoid the useless transcription of mRNAs, which would not be translated.

Finally, we demonstrated a good correspondence between the data we obtained and the translation efficiencies deduced from the ribosome profiling in vivo data [3,17,18].

To conclude this work, we demonstrated an approach to create and analyze Flow-seq libraries of reporter constructs with variable lengths and showed its suitability for assessing the influence of 5′-UTR on translation efficiency in the *E. coli* system.

## 4. Materials and Methods

### 4.1. Strains and Plasmids

Molecular cloning was carried out on the *E. coli* JM109 strain. The *E. coli* BL21 (DE3) strain was used in fluorescence-activated cell sorting. All strains were grown at 37 °C in LB medium (10 g/L tryptone, 5 g/L yeast extract, 5 g/L NaCl) or on LB agar plates supplied with 100 μg/mL ampicillin.

Plasmid pRFPCER [12] was used as a cloning vector to create a library according to a previously published protocol [6]. Plasmid T7-pRFPCER with T5-promoter before the *rfp* gene and T7 promoter before the *cer* gene served as internal controls with 5′-UTRs without variations.

### 4.2. Library Construction and Sorting

Oligonucleotides 5′-TAATACGACTCACTATAGGGN…NATGAAAGAGGACGAGAGC-3′ designed to contain the T7 promoter, natural 5′-UTR sequence and the first 18 nucleotides of the *cerulean* gene were used as single-stranded DNA templates. The library consisted of natural 5′-UTRs from the first genes of the operons *E. coli* BW25113 strain, with an inclusive length range of 2–60 nucleotides.

The double-stranded insertions were created by amplifying templates with primers 5′-GTGCTCAAGCTTTAATACGACTCACTATAGGG-3′ and 5′-CTGGCCGGATCCCCCGTCGCGGGTGCTCTCGTCCTCTTT-3′, which contained HindIII and BamHI restriction sites, respectively. Both the pRFPCER plasmid and library inserts were digested using HindIII and BamHI; then, inserts were ligated with linearized vectors and transformed into ultracompetent JM109 cells.

Plasmids were isolated by using a QIAprep Spin Miniprep Kit. The obtained library was transformed into BL21 (DE3) cells and grown on the LB agar plate with 100 μg/mL ampicillin at 37 °C overnight. After adding 2 mL of LB medium, colonies were scraped from the plate, centrifuged, and washed with PBS (140 mM NaCl, 10 mM phosphate buffer, 3 mM KCl, pH 7.4). Washed cells were resuspended in 1 mL PBS, diluted to ca 0.004 A600 and sorted with Becton Dickinson FACSAria III while monitoring CER fluorescence at 405/530 and RFP fluorescence at 561/582 nm.

The cells containing the plasmids library were sorted depending on the CER and RFP fluorescence intensity ratio in a logarithmic scale and collected into eight fractions. A total of 10,000 cells was sorted, which exceeded the diversity of the 5′-UTR library by more than 10 times. The number of cells collected for each fraction was proportional to the total number of cells with a certain CER/RFP fluorescence intensity ratio. Each fraction was grown in liquid LB medium with 100 μg/mL ampicillin at 37 °C overnight with vigorous shaking. Aliquots of 200 µL cells were taken from each fraction into a transparent 96-well plate. The cells were washed with 0.9% NaCl two times, centrifuged and resuspended in 200 µL 0.9% NaCl. The samples were transferred into a black 96-well plate and measured with a Perkin-Elmer Victor X5 reader using wavelengths of 531/595 nm for the RFP and 430/486 nm for the CER, using a fluorescence of 200 µL 0.9% NaCl as the background. After background subtraction, the CER/RFP fluorescence ratio was calculated and normalized to the ratio for the reference plasmid T7-pRFPCER. The 5′-UTR region was located before the *cerulean* gene, thus, the CER/RFP fluorescence ratio represented translation efficiency.

### 4.3. Sequencing

Cell suspensions obtained from eight fractions were used for plasmid isolation followed by PCR amplification with the primer pair 5′-GTGCTCAAGCTTTAATACGACTCACTATAGGG-3′ and 5′-GCTCTCGTCCGTCTCTTTC-3′. The PCR products were purified and checked with electrophoresis in 2% agarose gel. The obtained amplicons were used for the library preparation with the MGIEasy Universal DNA Library Prep (MGI Tech, Wuhan, China). DNA libraries were sequenced on the MGIseq-2000 (MGI Tech) utilizing the DNBSEQ-G400RS High-throughput Sequencing Set (cat. FCL PE100, MGI Tech, Wuhan, China) in the SB RAS Genomics Core Facility (ICBFM SB RAS, Novosibirsk, Russia).

### 4.4. Data Analysis

The raw PE reads were trimmed using cutadapt v4.0 (Marcel Martin, Stockholm, Sweden) [22] and merged with usearch v11.0.667 (Robert Edgar, Sonoma, USA) [23]. Merged reads were mapped to reference constructs using usearch. As a result, a table was generated, which reflected the representation of each type of 5-UTR in the eight analyzed libraries.

The distribution of reads between fractions corresponded to the Gaussian distribution with a pronounced peak. Thus, each of the variants was assigned the number of the fraction, in which the number of particular sequence reads was the maximum.

The fractions and their respective regions were divided into two groups based on the CER/RFP fluorescence ratio. The “ineffective” group of fractions and 5′-UTR regions included fractions A1–A4, for which the CER/RFP ratio, and, hence, the efficiency of translation, did not exceed that of the control plasmid. Fractions A5–A8, on the contrary, were found to be “effective”, because the CER/RFP ratio measured in them was higher than in the control plasmid.

The RNA hairpin folding free energy ΔG at 37 °C for a set of 5′-UTR appended with a constant 16 nt of ORF (ATGAAAGAGGACGAGA) was calculated using RNAfold [24]. RNAduplex v2.5.1 from the ViennaRNA package [21] was used to assess the effectiveness of the interaction of the anti-Shine–Dalgarno sequence (ACCTCCTT) with 5-UTR.

## Data Availability

Not applicable.

## Supplementary Materials

The following supporting information can be downloaded at: https://www.mdpi.com/article/10.3390/ijms232012293/s1.
