# Supplementary material for: Flow-Seq Evaluation of Translation Driven by a Set of Natural Escherichia coli 5′-UTR of Variable Length
Source: Int J Mol Sci. 2022 Oct 14;23(20):12293. doi: 10.3390/ijms232012293 (PMC9604319; doi:10.3390/ijms232012293)
Supplement: Supplementary file 1 [file ijms-23-12293-s001.zip › SI.pdf]

International Journal of *Molecular Sciences*

Supplementary information

Flow-seq evaluation of translation driven by a set of natural *Escherichia coli* 5′-UTR of variable length

Ekaterina S. Komarova[1], Anna N. Slesarchuk[2], Maria P. Rubtsova[2], Ilya A. Osterman[3], Alexey E. Tupikin[4], Dmitry V. Pyshnyi[4], Olga A. Dontsova[2,3,5,6], Marsel R. Kabilov[4*] and Petr V. Sergiev[1,2,3,6*]

[1] Institute of Functional Genomics, Lomonosov Moscow State University, 119992, Moscow, Russia
[2] Department of Chemistry, Lomonosov Moscow State University, Moscow, 119991, Russia
[3] Center for Life Sciences, Skolkovo Institute of Science and Technology, Skolkovo, Moscow region 143025, Russia
[4] Institute of Chemical Biology and Fundamental Medicine, Siberian Branch of the Russian Academy of Sciences, Novosibirsk, 630090, Russia
[5] Shemyakin-Ovchinnikov Institute of Bioorganic Chemistry, Russian Academy of Sciences, Moscow 119992, Russia
[6] Belozersky Institute of Physico-Chemical Biology, Lomonosov Moscow State University, Moscow 119992, Russia
* Correspondence: petya@genebee.msu.ru (P.V.S.) and kabilov@niboch.nsc.ru (M.R.K.)

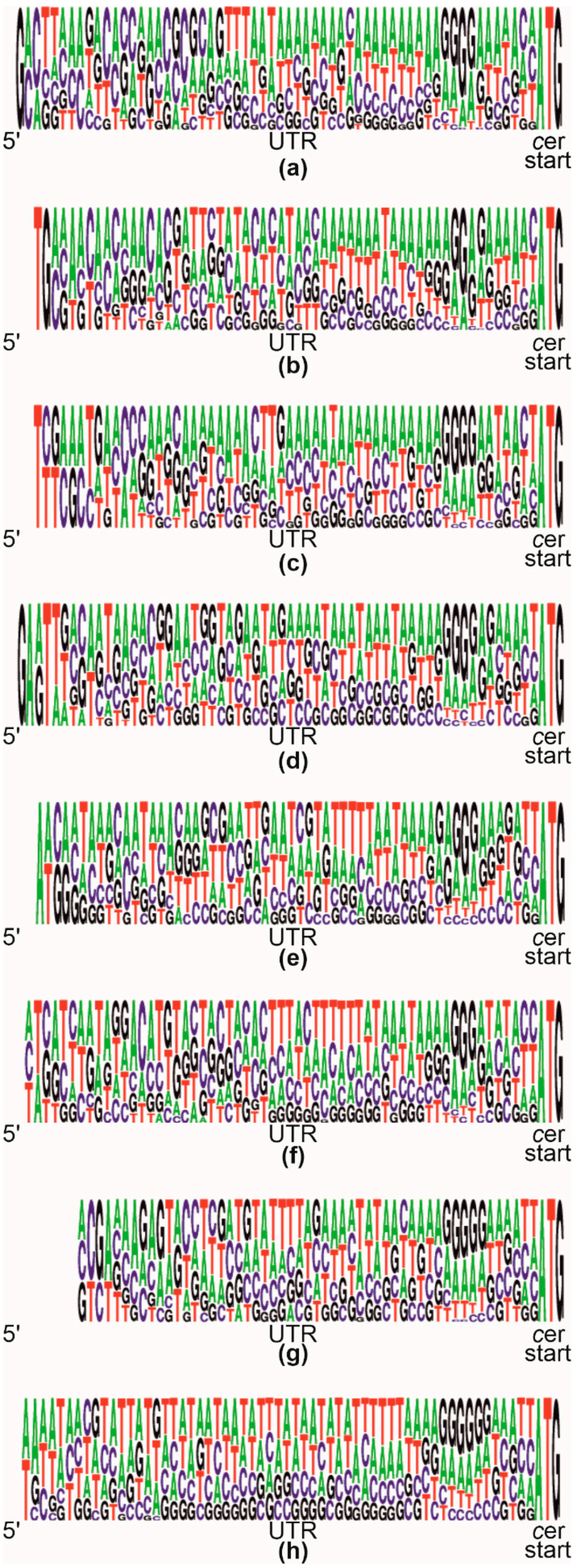

Supplementary Figure S1. Nucleotide composition of the 5′-UTR sequences sorted by translation efficiency shown as sequence logo [1]. (**a-h**) Bins from 1 to 8, ordered by increase in translation efficiency. The sequences are aligned by their 3′-edge (ATG start codon). Height of a letter represents an occurrence of particular nucleotide at particular position of the 5′-UTR.

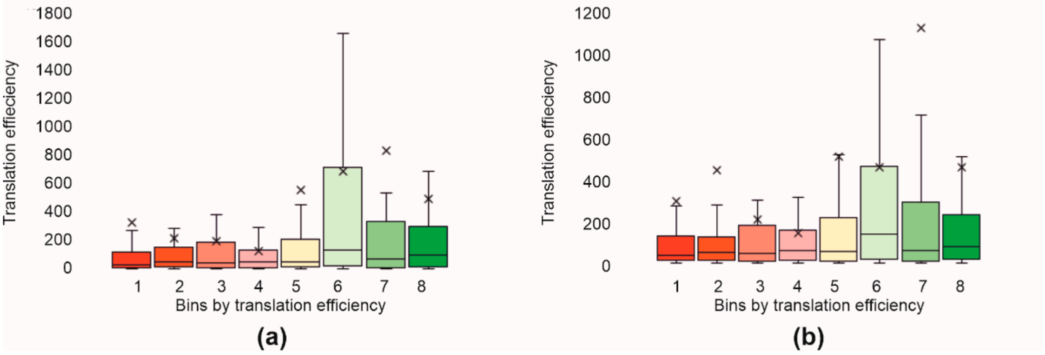

Supplementary Figure S2. Comparison of translation efficiencies observed by Flow-seq and those obtained by ribosome profiling. (**a**) Bins sorted by translation efficiency are marked below the bars. Y-axis corresponds to the translation efficiency determined by ribosome profiling in [2]. (**b**) The same as (a) for the ribosome profiling described in [3]. Quartile ranges are shown as solid bars (25% to 50%)

Supplementary references

1. Crooks, G.E.; Hon, G.; Chandonia, J.-M.; Brenner, S.E. WebLogo: A Sequence Logo Generator: Figure 1. *Genome Res.* **2004**, *14*, 1188–1190, doi:10.1101/gr.849004.
2. Elgamal, S.; Katz, A.; Hersch, S.J.; Newsom, D.; White, P.; Navarre, W.W.; Ibba, M. EF-P Dependent Pauses Integrate Proximal and Distal Signals during Translation. *PLoS Genet* **2014**, *10*, e1004553, doi:10.1371/journal.pgen.1004553.
3. Kannan, K.; Kanabar, P.; Schryer, D.; Florin, T.; Oh, E.; Bahroos, N.; Tenson, T.; Weissman, J.S.; Mankin, A.S. The General Mode of Translation Inhibition by Macrolide Antibiotics. *Proc Natl Acad Sci USA* **2014**, *111*, 15958–15963, doi:10.1073/pnas.1417334111.
